# Supplementary material for: Sustainability of Diets Consumed by UK Adolescents and Associations Between Diet Sustainability and Meeting Nutritional Requirements
Source: Nutrients. 2025 Jun 27;17(13):2140. doi: 10.3390/nu17132140 (PMC12251345; doi:10.3390/nu17132140)
Supplement: Supplementary file 1 [file nutrients-17-02140-s001.zip › nutrients-3702826-supplementary.pdf]

Supplementary Materials

Supplementary S1

A systematic disaggregation approach was undertaken as follows:

1. Items were grouped into categories (e.g. for composite dishes featuring wholegrains, these were grouped into 4 categories: wholemeal sandwiches, breakfast cereals, porridges and breakfast alternatives).
2. The five most commonly consumed items within these categories in the FUEL dataset were identified.
3. We sought to estimate the typical proportion each category contributed to the specified food group (e.g. the typical proportion a wholegrain sandwich contributed to the 'wholegrain' food group). This was done through searching supermarket websites for the five most commonly consumed items within the category. We reviewed ingredients lists to identify the proportion of the specified food group within the item (e.g. the proportion of wholegrain in a wholemeal ham sandwich). An average of the five values was calculated.
4. The resulting multiplicative factor was applied to all items in the group, resulting in estimates for the intake of the food group in grams.

Wholegrains:

Sandwiches:

**NDNS food code**	**Relevant items**	**Items used to calculate average:**	**Average wholemeal component:**
110 (cereal based savouries)	Bacon sandwich with wholemeal/oatmeal bread<br>Beef sandwich with wholemeal/oatmeal bread<br>BLT sandwich with wholemeal/oatmeal bread<br>Breakfast sandwich with wholemeal/oatmeal bread<br>CHICKEN AND BACON SANDWICH WITH WHOLEMEAL/OATMEAL BREAD<br>Chicken mayo sandwich with wholemeal/oatmeal bread<br>CHICKEN SALAD SANDWICH WITH MAYO WITH WHOLEMEAL/OATMEAL BREAD<br>Chicken salad sandwich with wholemeal/oatmeal bread<br>CORNED BEEF SANDWICH WITH WHOLEMEAL/OATMEAL BREAD<br>HAM SANDWICH WITH MAYO WITH WHOLEMEAL/OATMEAL BREAD<br>Ham sandwich with wholemeal/oatmeal bread<br>PRAWN MAYO SANDWICH WITH WHOLEMEAL/OATMEAL BREAD<br>SALMON AND CUCUMBER SANDWICH WITH WHOLEMEAL/OATMEAL BREAD<br>SMOKED SALMON AND CREAM CHEESE SANDWICH WITH WHOLEMEAL/OATMEAL BREAD<br>Tuna mayo sandwich with wholemeal/oatmeal bread<br>EGG MAYONNAISE SANDWICH WITH WHOLEMEAL/OATMEAL BREAD	Tesco Just Ham Sandwich<br>Sainsbury's Chicken Salad Sandwich<br>ASDA Tuna Mayo Sandwich<br>Tesco Cheese Triple Sandwich<br>Tesco Smoked Ham & Cheddar Sandwich	**63%**
29 (cheese dishes)	Cheese and ham sandwich with wholemeal/oatmeal bread<br>Cheese sandwich with wholemeal/oatmeal bread<br>PLOUGHMAN'S SANDWICH WITH WHOLEMEAL/OATMEAL BREAD		

	Welsh rarebit, on wholemeal (brown) toast		

Breakfast cereals - high percentage wholegrain:

**NDNS food code**	**Relevant items**	**Items used to calculate average:**	**Average wholemeal component:**
7 (other breakfast cereals)	All bran (including supermarket brands)<br>Bran flakes, supermarket brands<br>Oat flakes (e.g. Oatibix Flakes)<br>Nestle Shreddies<br>Shredded Wheat (e.g. Nestle)<br>Shredded Wheat, bitesize (e.g. Nestle)<br>Shreddies, supermarket brands (e.g. Tesco Malt Wheats)<br>Weetabix / Wheat biscuits<br>Weetabix crunchy bran<br>Weetabix, high protein<br>Kellogg's Bran Flakes	Weetabix/wheat biscuits<br>Nestle shreddies<br>Weetabix, high protein<br>Nestle Cheerios<br>Shreddies, supermarket brands (e.g. Tesco Malt Wheats)	**92%**
5 (sugar coated breakfast cereals)	Frosted Wheats/Shreddies (e.g. Kellogg's, Nestle)<br>Nestle Cheerios<br>Nestle Oat Cheerios (low sugar)		

Breakfast cereals - high percentage wholegrain with added items:

**NDNS food code**	**Relevant items**	**Items used to calculate average:**	**Average wholemeal component:**
7 (other breakfast cereals)	Fruit filled wheats (e.g. Kellogg's Raisin Wheats)<br>Fruit 'n' Fibre cereal, supermarket brands<br>Muesli with fruit & nuts (eg. Dorset cereals really nutty)<br>Muesli, Swiss style, no added sugar<br>Muesli, Swiss style, with added sugar (e.g. Alpen original)<br>Kellogg's Fruit 'n' Fibre cereal<br>Weetabix minis, chocolate<br>Weetabix minis, fruit and nut<br>Weetabix, chocolate	Chocolate hoops cereal (79)<br>Honey hoops cereal (e.g. Nestle Honey Cheerios) (59)<br>Weetabix minis, chocolate (31)<br>Weetabix, chocolate (19)<br>Fruit 'n' Fibre cereal, supermarket brands (15)	**80%**
5 (sugar coated breakfast cereals)	Chocolate hoops cereal (79)<br>Coco Shreddies (e.g. Nestle) (5)<br>Honey hoops cereal (e.g. Nestle Honey Cheerios) (59)		

Breakfast cereals - medium percentage wholegrain:

**NDNS food code**	**Relevant items**	**Items used to calculate average:**	**Average wholemeal component:**
7 (other breakfast cereals)	Crunchy oat cereal (granola) with chocolate/toffee (e.g. Sainsburys chocolate crisp)<br>Crunchy oat cereal (granola) with fruit (e.g. Tesco strawberry crisp)<br>Crunchy oat cereal (granola) with nuts (e.g. Jordans crunchy crisp with nuts)	Cookie crisp/crunch cereal (including Nestle, supermarket brands)<br>Nesquik chocolate cereal	**48%**

	Crunchy oat cereal (granola) without nuts (e.g. Quaker oat granola golden crunch)<br>Crunchy, cluster type cereal (e.g. Kelloggs/Nestle)<br>Gluten-free granola<br>Gluten-free muesli<br>Bran Flakes with sultanas / Sultana Bran (including supermarket brands)<br>Muesli with fruit only, no nuts (eg. Dorset cereals cherries and berries)<br>Special Flakes, with berries/fruit (e.g. Kellogg's Special K)<br>Kellogg's Special K (original)	Crunchy oat cereal (granola) without nuts (e.g. Quaker oat granola golden crunch)<br>Crunchy, cluster type cereal (e.g. Kelloggs/Nestle)<br>Crunchy oat cereal (granola) with chocolate/toffee (e.g. Sainsburys chocolate crisp)	
5 (sugar coated breakfast cereals)	Boulder type cereal (e.g. Nestle Golden Nuggets)<br>Cookie crisp/crunch cereal (including Nestle, supermarket brands)<br>Multigrain hoops (cheerios), supermarket brands<br>Nesquik chocolate cereal<br>Nestle Curiously Cinnamon<br>Special Flakes, with honey/chocolate (e.g. Kellogg's Special K)<br>Weetabix Weetos<br>Crunchy, cluster type cereal (e.g. Kelloggs/Nestle)		

Breakfast cereals - low percentage wholegrain:

**NDNS food code**	**Relevant items**	**Items used to calculate average:**	**Average wholemeal component:**
7 (other breakfast cereals)	Kellogg's Rice Krispie Mulitgrain Shapes<br>Special Flakes, supermarket brands	Kellogg's Rice Krispie Mulitgrain Shapes<br>Tesco Low Fat Special Flake Cereal	**26%**

Porridges:

**NDNS food code**	**Relevant items**	**Items used to calculate average:***	**Average wholemeal component:**
7 (other breakfast cereals)	Oats (uncooked)<br>Porridge, made with semi skimmed milk<br>Porridge, made with skimmed milk<br>Porridge, made with soya milk<br>Porridge, made with water<br>Porridge, made with whole milk<br>Porridge sachet, plain, made up with milk (e.g. Oat So Simple Original)<br>Porridge sachet, syrup/fruit flavour, made up with milk (e.g. Oat So Simple apple and blueberry)<br>Ready Brek, made up with semi skimmed milk<br>Ready Brek, made up with skimmed milk<br>Ready Brek, made up with whole milk	BBC Good Food recipe<br>Oat So Simple Original<br>Ready Brek	**14%**

*cooking instructions used

Breakfast alternatives:

**NDNS food code**	**Relevant items**	**Items used to calculate average:**	**Average wholemeal component:**

8 (breakfast alternatives)	Belvita breakfast biscuits (45)<br>Belvita soft bakes (breakfast biscuits) (11)<br>Cereal bar with fruit and nuts, with or without coating (44)<br>Cereal bar with fruit, with or without coating (37)<br>Cereal bar with nuts, with or without coating (15)<br>Cereal bar, low calorie (e.g. Alpen light bar) (17)<br>Cereal bar, plain (103)<br>Chewy cereal bar (e.g. Harvest chewee) (9)<br>Kellogg's Nutrigrain bar (5)<br>Special K cereal bar (20)<br>Toddler cereal bar (e.g. Organix carrot cake cereal bar) (1)	Belvita Milk & Cereal Biscuits<br>Belvita Soft Bakes Chocolate Chip<br>Nature Valley Fruit & Nut Cereal Bars<br>Kellogg's Special K Milk Chocolate Bars<br>Organix carrot cake cereal bar	**29%**

Potato:

Instant potato:

**NDNS food code**	**Relevant items**	**Items used to calculate average:**	**Average potato component:**
65 (other potatoes)	Instant potato (e.g. Smash), made up with milk<br>Instant potato (e.g. Smash), made up with water	Smash The Original Instant Mash Potato	**17%**

High percentage potato items:

**NDNS food code**	**Relevant items**	**Items used to calculate average:**	**Average potato component:**
65 (other potatoes)	Cheesy mash	ASDA Savoury & Smooth Cheesy Mash<br>Tesco Finest Baby Potato Salad<br>ASDA Reduced Fat Potato Salad<br>Tesco Fresh Potato Gnocchi<br>Hearty Food Co Potato Croquette 700G	**77%**
66 (potato products)	Gnocchi<br>Potato croquettes<br>Potato fritters, fried		
72 (dressed salad dishes)	Potato salad, with mayonnaise/salad cream<br>Potato salad, with reduced fat mayonnaise/salad cream		

Medium percentage potato items:

**NDNS food code**	**Relevant items**	**Items used to calculate average:**	**Average potato component:**
56 (Fish dishes)	Fisherman's pie, home made	Tesco Indian Saag Aloo 300G<br>Kirstys Lentil Cottage Pie<br>ASDA Cottage Pie<br>ASDA Shepherd's Pie<br>ASDA Extra Special Creamy Dauphinoise Potatoes	**47%**
65 (other potatoes)	Spinach and potato curry (e.g. saag aloo)<br>Potato curry, with tomatoes<br>Dauphinoise potatoes<br>Potato gratin (cheese & potato bake)<br>Potato bake, with cream and bacon<br>Bubble and squeak		
71 (vegetable dishes)	Pea and potato curry (e.g. aloo matar)<br>Vegetarian cottage pie, vegetable-based		

72 (dressed salad dishes)	Aubergine & potato curry (e.g. brinjal aloo)<br>Cauliflower & potato curry (e.g. aloo gobi)		
108 (meat and potato dishes)	Cottage pie (beef), home made<br>Cumberland pie<br>Shepherd's pie (lamb), home made		

Dairy:

High percentage dairy:

NDNS food code	Relevant items	Items used to calculate average:	Average dairy component:
17 (milk based sweet puddings)	Condensed milk<br>Confectioners custard<br>Custard, low fat<br>Custard, made with sugar (homemade)<br>Custard, ready to eat, individual pots<br>Custard, ready to serve<br>Instant mousse dessert (made with milk) (e.g. Angel Delight)<br>Rice pudding, with fruit<br>Rice pudding / semolina, canned<br>Rice pudding, low fat, ready to eat, not canned (e.g. Muller rice)<br>Rice pudding, not canned	Ambrosia Ready To Eat Devon Custard 4 X 125G<br>Ambrosia Custard 400G<br>Angel delight - 300ml milk for 59g sachet<br>Ambrosia Light Less Sugar & Fat Rice Pudding 4X125g<br>Ambrosia Creamed Rice Pudding 4 X 125G	**76%**
25 (cream)	Dream topping		

Medium percentage dairy:

NDNS food code	Relevant items	Items used to calculate average:	Average dairy component:
17 (milk based sweet puddings)	Cornmeal porridge<br>Crème brulee<br>Trifle	GRACE INSTANT CORNMEAL H/STYLE PORRIDGE<br>Tesco Creme Brulee<br>Tesco Strawberry Trifle 600G<br>BBC good food recipe details<br>Tesco Sour Cream & Chive Dip 200G	**51%**
25 (cream)	Butter icing<br>Sour cream based dips		

Yoghurt/fromage frais with added items (e.g. fruit, cereal):

NDNS food code	Relevant items	Items used to calculate average:	Average dairy component:
18 (low fat yoghurt)	Fruit yoghurt, low fat<br>Fruit yoghurt, very low fat, with added sugar<br>Fruit yoghurt, virtually fat free, with artificial sweetener (e.g. Muller Light)<br>Greek-style yoghurt, whole milk, fruit<br>Low fat hazelnut yoghurt<br>Probiotic yoghurt drink with fruit (e.g. Actimel)<br>yoghurt, greek style, fruit/honey	Ski Variety Fruit Yogurt 4X120g<br>Danone Actimel Strawberry Drink<br>Frubes Strawberry Red Berry & Peach Yogurts<br>Onken Mango & Passion Fruit Yogurt 450G	**92%**

	Yoplait Frubes/Wildlife Choobs	Muller Corner Strawberry Peach & Apricot Yogurts	
19 (whole milk yoghurt)	Children's yoghurt drink (e.g. Munch bunch squashums yoghurt drink)<br>Fruit yoghurt<br>Greek-style yoghurt, whole milk, fruit<br>Petit Filous fromage frais<br>Yoghurt, twin pot with coated cereal/crumble (e.g. Muller Corner)<br>Yoghurt, twin pot with fruit (e.g. Muller Corner)		

Milkshakes:

NDNS food code	Relevant items	Items used to calculate average:	Average dairy component:
24 (milkshakes)	Milkshake / Milk drink, not chocolate (e.g. strawberry, banana)<br>Milkshake made with powder and milk, chocolate<br>Milkshake made with powder and milk, fruit flavoured<br>Milkshake ready to drink (e.g. Frijj, yazoo)<br>Milkshake, thick, with ice cream, purchased (e.g. McDonald's, Wimpy)<br>Weetabix on the go, breakfast drink	Tesco Strawberry Flavoured Milk 1L<br>Nesquik Chocolate Powder 500G<br>Nesquik Strawberry Powder 500G<br>Frijj Strawberry 400Ml<br>Weetabix On The Go Vanilla Drink 250Ml	**87%**

Spreadable butter:

NDNS food code	Relevant items	Items used to calculate average:	Average dairy component:
30 (butter)	Spreadable butter, unsalted	Lurpak Unsalted Spreadable Blend of Butter and Rapeseed Oil 400g<br>Lurpak Lighter Spreadable Blend of Butter and Rapeseed Oil 400g<br>Lurpak Slightly Salted Spreadable Blend of Butter and Rapeseed Oil 400g	**56%**
31 (dairy fat spreads)	Reduced fat spreadable butter (e.g. Lurpack Light)<br>Spreadable butter, salted		

Ice cream with low/no added components:

NDNS food code	Relevant items	Items used to calculate average:	Average dairy component:
79 (ice cream)	Frozen yoghurt<br>Ice cream, reduced fat<br>Mini milk ice lolly<br>Premium vanilla ice cream (e.g. Mackies, Haagen Daz)<br>Soft scoop chocolate ice cream<br>Soft scoop Ice cream, flavoured (e.g. raspberry ripple)<br>Soft scoop vanilla ice cream	Mackies Traditional Luxury Dairy Ice Cream 1 Litre<br>Wall's Ice Cream Multipack (mini milk)	**72%**

Ice cream with added items (e.g. cone, chocolate):

NDNS food code	Relevant items	Items used to calculate average:	Average dairy component:
79 (ice cream)	Artic roll<br>Cornetto/king cone (Including supermarket brands)<br>Feast ice cream<br>Fruit lolly with ice cream filling (e.g. Ice cream split)<br>Ice cream dessert (e.g. Viennetta)<br>Premium ice cream with chocolate/caramel/nuts/biscuit (e.g. Ben and Jerry's)<br>Sundae dessert<br>McDonald's Mcflurry<br>Mr whippy style ice cream<br>Choc ice<br>Chocolate mousse/dessert, low fat<br>Magnum classic or white	Cornetto Classic Ice Cream Cones 6X90ml<br>Feast Chocolate Ice Cream 4 X 90Ml<br>Viennetta Vanilla Ice Cream Dessert 650Ml<br>Ben & Jerry's Cookie Dough Vanilla Ice Cream 465Ml<br>Magnum Classic Ice Creams 6 X 100Ml	**49%***

*average of 'added items' component calculated & subtracted from average for 'ice cream with low/no added components'

Egg:

High percentage egg:

NDNS food code	Relevant items	Items used to calculate average:	Average egg component:
38 (egg dishes)	Egg mayonnaise sandwich filler	Tesco Egg Mayonnaise Sandwich Filler	**74%**

Medium percentage egg:

NDNS food code	Relevant items	Items used to calculate average:	Average egg component:
38 (egg dishes)	Omelette with ham & cheese<br>Omelette with meat (e.g. ham)<br>Omelette, with cheese<br>Omelette, with vegetables<br>Egg curry<br>Egg mayonnaise sandwich<br>Scotch egg (including mini/picnic size)	Hearty Food Co. 2 Cheese Omelettes<br>Tesco 2 Med Vegetable Omelettes 200g<br>Tesco Egg & Cress Sandwich<br>Tesco 4 Scotch Eggs	**42%**

Low percentage egg:

NDNS food code	Relevant items	Items used to calculate average:	Average egg component:
38	Eggy bread/french toast<br>Fish/seafood quiche (e.g. salmon)<br>Meat based quiche (e.g. quiche lorraine)<br>Meat based quiche (e.g. quiche lorraine), reduced fat<br>Vegetable based quiche (e.g. cheese & onion)	Sainsbury's Salmon, Broccoli & Cheddar Quiche, Taste the Difference<br>Sainsbury's Quiche Lorraine<br>Sainsbury's Cheese & Onion Quiche	**10%**

Supplementary S2

Construction of PHD index score.

Characteristics of PHD recommendations	Food groups	Scoring
Emphasised foods with a non-zero lower limit.	Vegetables, fruits and unsaturated oils.	0 points: <50% of lower limit.<br>1 point: 50% - 100% of lower limit.<br>2 points: lower limit - target value.<br>3 points: > target value.
Emphasised foods with zero lower limit.<br>Emphasised food with single target value (lower limit not specified).	Wholegrains, fish, legumes and nuts	0 points: <25% of target value.<br>1 point: 25%-50% of target value.<br>2 points: 50%-100% of target value.<br>3 points: >target value.
Restricted foods.	Potatoes, dairy, beef, lamb and pork, chicken, eggs.	0 points: >150% of upper limit.<br>1 point: 100%-150% of upper limit.<br>2 points: target value to upper limit.<br>3 points: <target value.
Restricted foods, but target value same as upper limit.	All sweeteners.	50% of upper limit applied as new target value, then same principle as for other restricted foods applied.

Supplementary S3

Sensitivity analysis: after excluding observation >3 SDs from the mean.

		**Unadjusted MD (CI), P-value**	**P-value**
% RNI	Iron	-2.4 (-2.9, -2.0)	<0.001
	Calcium	-2.8 (-3.5, -2.2)	<0.001
	Vitamin B12	-13.7 (-16.2, -11.2)	<0.001
	Vitamin D	-0.6 (-0.8, -0.4)	<0.001
	Zinc	-2.9 (-3.3, -2.4)	<0.001
	Iodine	-2.4 (-3.0, -1.7)	<0.001
% RI	Fibre	-0.2 (-0.6, 0.1)	0.2
% TEI	Carbohydrates	0.4 (0.3, 0.5)	<0.001
g	Omega-3	-0.04 (-0.06, -0.03)	<0.001
	Protein	-2.6 (-2.9, -2.2)	<0.001
